# Healing mechanisms of Galectin-1 in the ischemic heart

**DOI:** 10.1007/s12013-026-02069-1

**Published:** 2026-04-11

**Authors:** Natalie Y. Luo, Chisom Ovuegbe, Jijun Huang, Finosh G. Thankam

**Affiliations:** 1 Department of Translational Research, College of Osteopathic Medicine of the Pacific, Western University of Health Sciences, Pomona, CA 91766 USA; 2 Division of Endocrinology, Diabetes and Metabolism, Department of Medicine, David Geffen School of Medicine, University of California-Los Angeles, Los Angeles, CA 90095 USA

**Keywords:** Galectin-1, Myocardial infarction, Cardioprotection, Myocardial healing, Heart repair

## Abstract

Limited ability of the heart to regenerate aggravates myocardial infarction (MI), a major cause of heart failure. Galectin-1, an endogenous β-galactoside-binding lectin, accelerates heart regenerative and repair responses following MI by modulating immunological responses, promoting angiogenesis, and improving cell survival. Galectin-1 is associated with cardiomyocytes (CMs) survival by activating the PI3K/AKT signaling pathway and facilitates limited adverse remodeling by switching to M2 macrophages. The upregulation of Galectin-in response to ischemia-induced inflammation and hypoxia highlights its function in endogenous defense systems. Galectin-1 is critical in cardiac regeneration due to the synergistic immunomodulatory, angiogenic, and electrophysiological effects. Even though recent reports highlight importance of Galectin-1 in cardio protection, mechanistic investigations, and translational/clinical studies are limited. This article focuses on a comprehensive review on the current understanding regarding the cardioprotective effects and translational opportunities of Galectin-1 for the management of MI.

## Introduction

Heart failure (HF) is one of the dominant causes of mortality worldwide, with a projection of causing 35.6 million deaths in 2050 [1]. Myocardial infarction (MI) is the most common cause of heart failure. Pathologically, cardiac cells especially the cardiomyocytes (CMs) deteriorate due to the lack of oxygen leading to irreversible damage and decreased cardiac output [2]. Damage to the heart following MI triggers inflammatory and wound-healing responses resulting in scar formation. Inherently, the heart has very limited regenerative capacity, so repetitive infarctions and prolonged inflammatory responses increases the risk for HF [3]. Surgery and neurohormonal activation preserve the surviving CMs. Although surgical revascularization significantly improves clinical outcomes, restoring blood flow to previously ischemic myocardium induces ischemia-reperfusion injury, a process that modern cardioprotective strategies aim to minimize. Moreover, neurohormonal activation, which is otherwise protective, is short-lived thereby persisting in the damage [4, 5]. When cardiac function is beyond repair, heart transplantation is the last resort. Unfortunately, the current treatment approaches fail to address the loss of around 1 billion CMs following a massive MI [6].

Current regenerative strategies, including cell therapy and activation of endogenous cardiac progenitor cells, offer potential benefits in promoting cardiac repair. However, these approaches fail to fully restore heart function, primarily due to challenges in cell survival, integration, and long-term efficacy. For instance, skeletal myoblasts, were shown to recover myocardium providing functional improvement; however, failed to establish electrical connectivity with the host myocardium [7]. Clinical trials showed limited efficacy of skeletal myoblasts and high risk of arrhythmia due to limited gap junctions [7]. Interestingly, embryonic stem cells (EBCs) demonstrated stable grafts and electrical integration with host myocardium in guinea pig myocardial injury model [8]. However, the concern of the longevity and immunosuppression post-administration in humans offer significant challenge [6].

Cell signaling pathways are critical in regulating pathophysiological conditions and manipulating the molecular targets to restore the heart exhibit regenerative promise. On this context, Galectin-1 has been identified as a promising protein candidate due to its critical role in immunomodulation and tissue repair. Galectin-1 promotes angiogenesis, limited adverse remodeling, and cell survival, activating cardiac healing [9–11]. Notably, Galectin-1 interacts with endothelial cells to assist in the development and stabilization of new blood vessels and induces myofibroblast differentiation to increase production of collagen and ECM proteins [9]. Also, Galectin-1 is a key immunosuppressor by suppressing pro-inflammatory cytokines, encouraging the apoptosis of activated T-cells, and supporting tolerogenic dendritic cells [10, 11] in diverse diseases including MI. Despite these pro-healing effects, the implications of Galectin-1 in translational cardiology remain under-explored. On this juncture, this article focuses on a comprehensive review on the current understanding regarding the cardioprotective effects and translational opportunities of Galectin-1 for the management of MI.

## Biology of Galectin-1

**Fig. 1:**
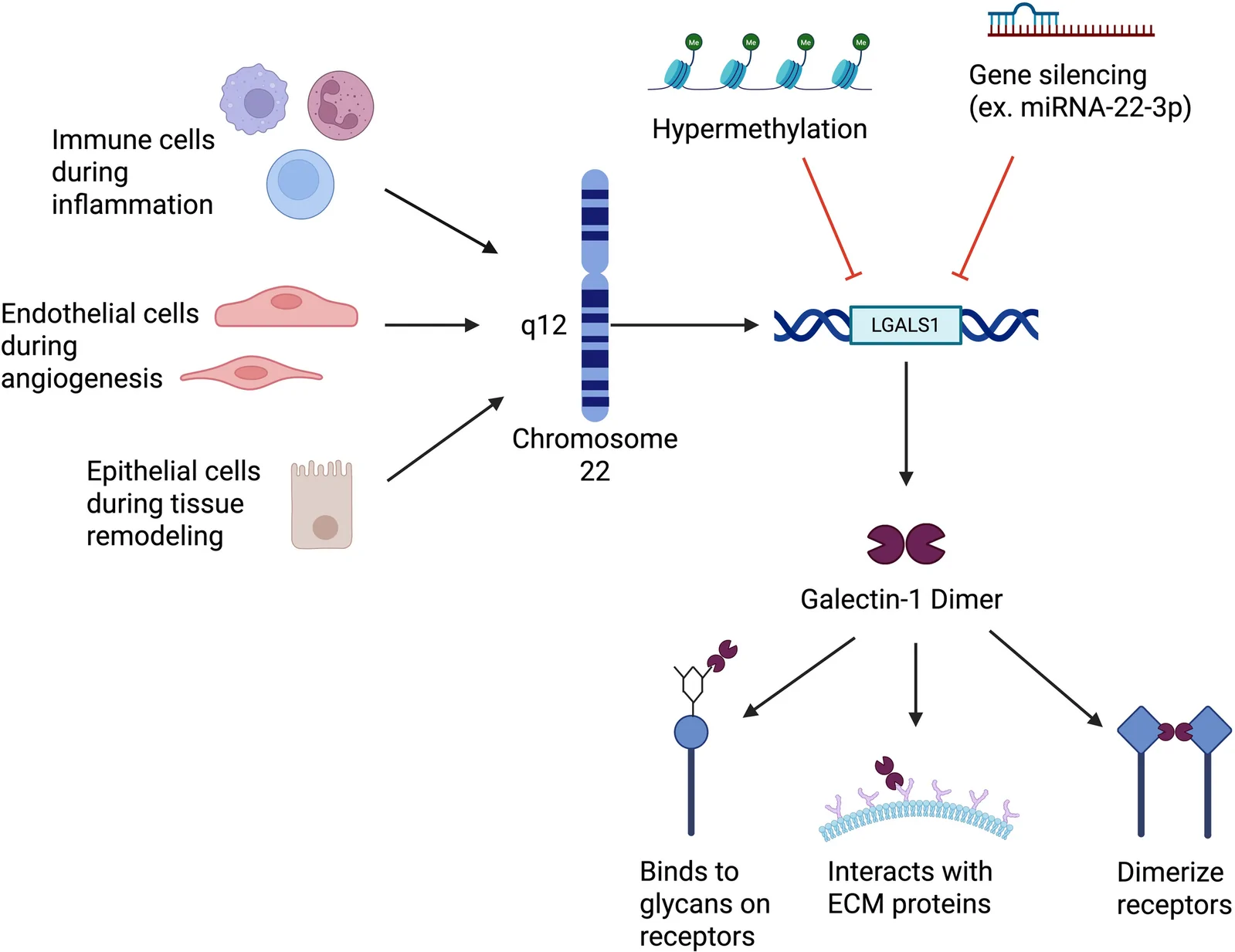
Biology and regulation of Galectin-1 expression and function: Schematic representation of the molecular biology, regulation, and functional interactions of Galectin-1. The LGALS1 gene, located on chromosome 22q12, is expressed in immune, endothelial, and epithelial cells during inflammation, angiogenesis, and tissue remodeling. Its expression is regulated by epigenetic mechanisms, including promoter hypermethylation and microRNA-mediated silencing (e.g., miRNA-22-3p). Galectin-1 is produced as a homodimer, which enables binding to β-galactoside-containing glycans on cell surface receptors and interactions with extracellular matrix proteins. These interactions facilitate receptor clustering and signaling, supporting its roles in immune modulation and tissue repair

Galectin-1 is encoded by the LGALS1 gene on chromosome 22q12 and is the first protein discovered in the galectin family (Fig. 1). Galectins are a type of lectin with a highly conserved carbohydrate recognition domain (CRD) that specifically binds to β-galactose-containing oligosaccharides [12]. LGALS1 gene is a relatively small single exon gene that directly encodes Galectin-1. The Galectin-1 protein is a homodimer of 14-kDa subunits, arranged in a sandwich-like structure with two anti-parallel β-sheets. LGALS1 has two galactoside-binding sites per dimer and is expressed in various tissues in normal and pathological conditions [13]. Galectin-1 is mostly found in the cytoplasm or secreted into the extracellular space, cell membrane and in the nucleus to regulate various nuclear processes, such as gene expression and cell cycle progression. Overall, Galectin-1 has membranous, intracellular and extracellular effects due to their ability to bind to multiple ligands associated with cell cycle, immunity, and inflammation [14, 15].

Galectin-1 interacts with various glycoconjugate ligands of the ECM, such as laminin and fibronectin, and those on endothelial cells, such as ROBO4 and CD36, and on T-lymphocytes. Human Galectin-1 is a dimer in solution that is stabilized by the monomer interaction at their junction and the highly conserved hydrophobic core [16]. A defining characteristic of the homodimeric Galectin-1 protein is its spontaneous dissociation into a monomeric form at low concentrations (Kd ~ 7 µM). The dissociated monomer binds to carbohydrates with lesser affinity [17]. The intracellular activity of Galectin-1 and its oxidized form is not reliant on its lectin function, whereas its extracellular activity relies on it [18, 19]. Each Galectin-1 monomer has six cysteine residues per subunit, which makes Galectin-1 highly sensitive to oxidation [20, 21]. Galectin-1 loses the lectin function following oxidation confirming cystine -SH residues are critical to maintain lectin activity. A unique characteristic of Galectin-1 is its secretion into the extracellular space, despite the absence of the signaling sequences required for the normal endoplasmic reticulum/Golgi pathway [22]. Extracellular Galectin-1 is slightly larger (~ 15 kDa), indicating the post-translational modifications pre- or post-secretion [23]. Also, Galectin-1 secretion depends on the functional interactions between the lectin and β-galactoside-containing cell surface receptors [24].

Galectin-1 is highly expressed in immunologically protected, activated immune cells, and endothelial cells, epithelial cells and is heavily involved in cardiac embryogenesis and vascular development [9, 25]. Ischemic heart disease and heart failure conditions, such as hypoxia, oxidative stress, and inflammatory injury, create tissue microenvironments that upregulate Galectin-1 [26, 27]. Hypermethylation of the Galectin-1 promoter site silences the LGALS1 gene and blocks transcription factors to decrease Galectin-1 expression [28].

### Regulation of Galectin-1

Galectin-1 expression is influenced by genetic, epigenetic and physiological/pathological conditions including hypoxia. Expression of the LGALS1 gene is driven by the degree of methylation and activity of transcriptional factors, such as AP1, NF-kB, and HIF-1 [27, 29–35]. Additionally, miRNA-22-3p, a biomarker for chronic heart failure, downregulates Galectin-1 expression by interfering with its mRNA [36, 37]. In addition, a single point mutation in the nek8 gene showed upregulation of Galectin-1 [38]. H-Ras, insulin (Dexamethasone, Methylisobutylxanthine, Insulin cocktail), and lipopolysaccharide (LPS) increases Galectin-1 expression. When Galectin-1 forms a complex with H-Ras, Galectin-1 stabilizes membrane anchoring, which then increases activity of Galectin-1 by a feed-forward loop of the Ras pathway [39]. Insulin indirectly increases Galectin-1 levels in adipose tissue through adipocyte differentiation [32]. LPS increases Galectin-1 expression by activating the TLR4 signaling pathway, which then activates NF-kB, a transcription factor that binds to LGALS1 and induces the expression of Galectin-1 [40, 41]. Hypoxia upregulates galectin-1 through the hypoxia-inducible factor (HIF) 1alpha protein [29] which is parallel to CMs where Galectin-1 expression is increased in acute MI and large artery atherosclerotic stroke [42, 43].

**Fig. 2:**
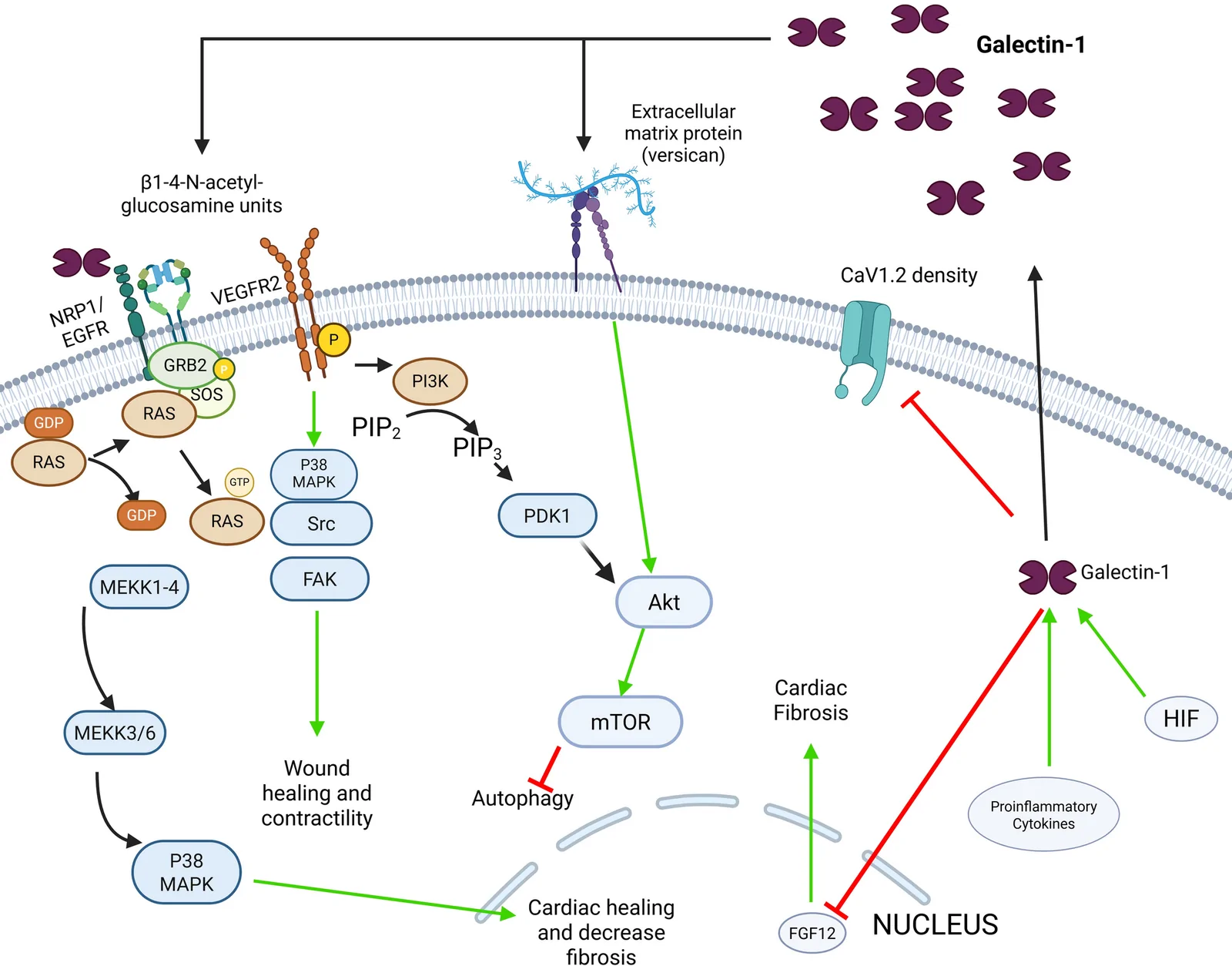
Regulation of Galectin-1 and its downstream signaling pathways in cardiac cells: Galectin-1 expression is induced by hypoxia (via HIF) and inflammatory cytokines in the cardiac microenvironment. Extracellular Galectin-1 binds glycosylated receptors (e.g., VEGFR2/NRP1) and extracellular matrix proteins, activating MAPK, VEGF-A, and PI3K/AKT/mTOR signaling pathways that promote angiogenesis, cardiac repair, and inhibition of autophagy. Intracellularly, Galectin-1 reduces CaV1.2 channel density and modulates nuclear signaling (e.g., FGF12), attenuating hypertrophy and fibrosis. These coordinated pathways position Galectin-1 as a key regulator of cardiac adaptation to stress

Downregulation of Galectin-1 involves transcription repression, enzymatic control, and post-translational inhibition. KLF12 is a transcription factor that directly binds to LGALS1 promoter region and reduces transcriptional activity of Galectin-1 [44]. High degree of methylation in CpG sites within the LGALS1 promoter region decreases expression of Galectin-1 [45]. Suppression of superoxide dismutase (SOD1), an antioxidant enzyme, changed cellular redox states and indirectly suppress Galectin-1 levels [46]. Small molecular inhibitors, such as OTX008, bind to Galectin-1, post-translationally, and block its lectin activity, preventing cell communication and immune modulation [47]. These mechanisms of action highlight the numerous regulatory strategies for Galectin-1 function which require further validation for cardiac applications. Overall, regulatory mechanism of Galectin-1 is depicted in Fig. 2.

## Extracellular and intracellular actions of Galectin-1

Galectin-1 has been shown to bind to multiple galactose-β1-4-N-acetyl-glucosamine (N-acetyl-lactosamine; LacNAc) units present on the branches of N- or O-linked glycans on a variety of cell surface receptors, such as CD2, CD3, CD7, CD43, CD95, CD45, CD44, CD97, GPC1, CD43, CD69, pre-BCR, and vascular endothelial growth factor receptor 2 (VEGFR2) [48–50]. This binding modulates a wide variety of processes, including segregation, endocytosis, and signaling pathways (Table 1). Additionally, Galectin-1 interacts with ECM proteins such as vasorin (Vasn), fibulin-2 (Fbln-2), Notch-2, lactadherin (Mfgm), and versican (Cspg-2) [51]. Owing to the broad binding capacity, Galectin-1 has been referred as a “jack of all trades,” capable of engaging with a wide spectrum of receptors and producing context-dependent effects [52].

Galectin-1 enhances epithelial-mesenchymal transition (EMT) through activation of the MAPK/JNK/p38 signaling cascade [53]. In intrauterine adhesion models, Galectin-1 acts via the JAK/STAT signaling pathway and promotes macrophage polarization toward the anti-inflammatory M2 phenotype, playing a vital role in TNF-α-mediated mesenchymal stem cell immunosuppression [54–57]. Galectin-1 interacts with FGF12 protein core within the nucleus, blocking FGF12 secretion and inhibiting the assembly of ribosomes that contain FGF12 [58]. In T cell modulation, Galectin-1 has been implicated in targeting glycoprotein surface receptors resulting in a wide range of immune effects, including impaired T cell adhesion and reduced rolling and capture during T cell migration [59]. Interestingly, Galectin-1 facilitates interactions between various cells and ECM and restrict vascular smooth muscle cell migration [60]. Also, Galectin-1 upregulation promotes angiogenesis [61].

**Table 1:** Summary of the intracellular and extracellular effects of Galectin-1

Category	Mechanism	Description	Reference
Intracellular	Pre-mRNA Splicing	Modulates CaV1.2 channel splicing via interaction with splicing complexes	Fan et al. (2019)
	Calcium Channel Regulation	Reduces CaV1.2 channel density on cardiomyocytes via splice variant-specific control	
	Intracellular Signaling	Activates MAPK/JNK/p38 pathway to enhance EMT and metastasis in ovarian cancer Activates JAK STAT and induces M2 macrophage polarization in intrauterine adhesion	Ou et al. (2022) Li et al. (2022)
	Nuclear Interaction	Binds to FGF12 → inhibits its secretion and ribosome assembly	Gędaj et al. (2024)
	mRNA-Binding Activity	Preferentially binds near stop codons with UGCA/UGGA and GAGCAG as binding motifs	Wei et al. (2021)
Extracellular	Glycan Binding to Cell Surface Receptors	CD45, CD44, CD97, GPC1, CD43, CD69, pre-BCR, VEGFR2	de la Fuente et al. (2014)
	Interaction with ECM proteins	Vasorin (Vasn), fibulin-2 (Fbln-2), Notch-2, lactadherin (Mfgm), versican (Cspg-2)	Vilen et al. (2021)
	Immune Modulation	Binds CD2, CD3, CD7, CD43, CD45, CD69, CD95 → impairs T cell adhesion, rolling, and migration	Norling et al. (2007)
	Cell-ECM Dynamics	Restricts vascular smooth muscle cell migration	Tsai et al. (2018)
	Angiogenesis in Tumors	Promotes blood vessel formation, e.g., in prostate cancer microenvironment	Thijssen et al. (2006)

## Emerging trends in Galectin-1 driven heart protecting mechanisms- - A bird’s eye view

Galectin-1 drives several critical signaling pathways that influence its cardioprotective effects. Activation of P38 mitogen-activated protein kinase (MAPK) pathway is known to promote cardiac healing through activating ADAM17 and prevent fibrosis through suppressing TGF-β induced SMAD2/3 phosphorylation [62, 63]. Galectin-1 influences the phosphatidylinositol 3-kinase (PI3K)/protein kinase B (AKT) pathway following ischemic injury. Activating the PI3K/AKT signaling and the downstream mammalian target of rapamycin (mTOR) mitigates MI and reperfusion injury by inhibiting autophagy [64, 65]. Hence, Galectin-1 demonstrates the potential to promote cell survival in cardiac tissue following the pathology (Tables 2 and 3).

Vascular endothelial growth factor (VEGF) pathway for blood vessel formation and the Wnt/β-catenin and epithelial-mesenchymal transition (EMT) pathways for myocardial growth as well as coronary vessel development are critical in cardiac survival [66, 67]. CMs express VEGF-A receptors, VEGFR1 and VEGFR2, on the cell surface, which allow VEGF-A to promote wound healing and contractility of the heart [68]. VEGF-A has also been shown to have profound angiogenic effects and improve systolic function following MI [69–71]. Interestingly, tumors treated with anti-VEGF-A antibodies, such as bevacizumab, secrete Galectin-1 promoting angiogenesis, demonstrating the significance of Galectin-1 in the VEGF-A pathway [72, 73]. The Wnt pathway plays a crucial role in cardiac development and improving cardiac function following myocardial injury [66, 74, 75]. Galectin-1 inhibition demonstrated decreased β-catenin activity in esophageal squamous cell carcinoma [76]; warranting further research to assess the effect in cardiac tissue. Additionally, EMT plays a significant role in myocardial growth and coronary vessel development. EMT mRNAs and genetic markers, such as SNAI2, Slug, and Snail are involved in mammalian annulus fibrosis and coronary artery disease [67, 77]. Galectin-1 activates the EMT pathway as shown in breast cancer and hepatocellular carcinomas [36, 78]. Their role of Galectin-1 in promoting cardiac differentiation and vascular development warrants further investigations.

**Table 2:** Cardioprotection pathways that can be affected by Galectin-1

Signaling Pathway	Cardioprotection	Role of Galectin-1	Reference
P38 mitogen-activated protein kinase (MAPK)	Increase cardiac healing and stops fibrosis	Enhances pathway to encourage cellular growth	Meijles et al. (2020) Chen et al. (2023)
Phosphatidylinositol 3-kinase (PI3K)/Protein kinase B (AKT)	Inhibits autophagy that mitigates myocardial infarction	Promotes pathway to increase cellular proliferation and survival	Guan et al. (2020) Popov et al. (2023)
Vascular endothelial growth factor (VEGF)	VEGF-A enhances wound healing and cardiac contractility	Anti-VEGF-A increases galectin-1 secretion to increase angiogenesis and cellular growth	Rios-Navarro et al. (2021) Pérez Sáez et al. (2021)
Wingless-related integration (Wnt)	Cardiac development and function	Inhibition decreased stem cell properties activated by the pathway	Zhang et al. (2022) Zhou et al. (2024)
Epithelial-Mesenchymal Transition (EMT)	Myocardial growth and coronary vessel development	Activate pathway for cellular growth	Zhou et al. (2010) Bacigalupo et al. (2015)

## Post-MI recovery

**Fig. 3:**
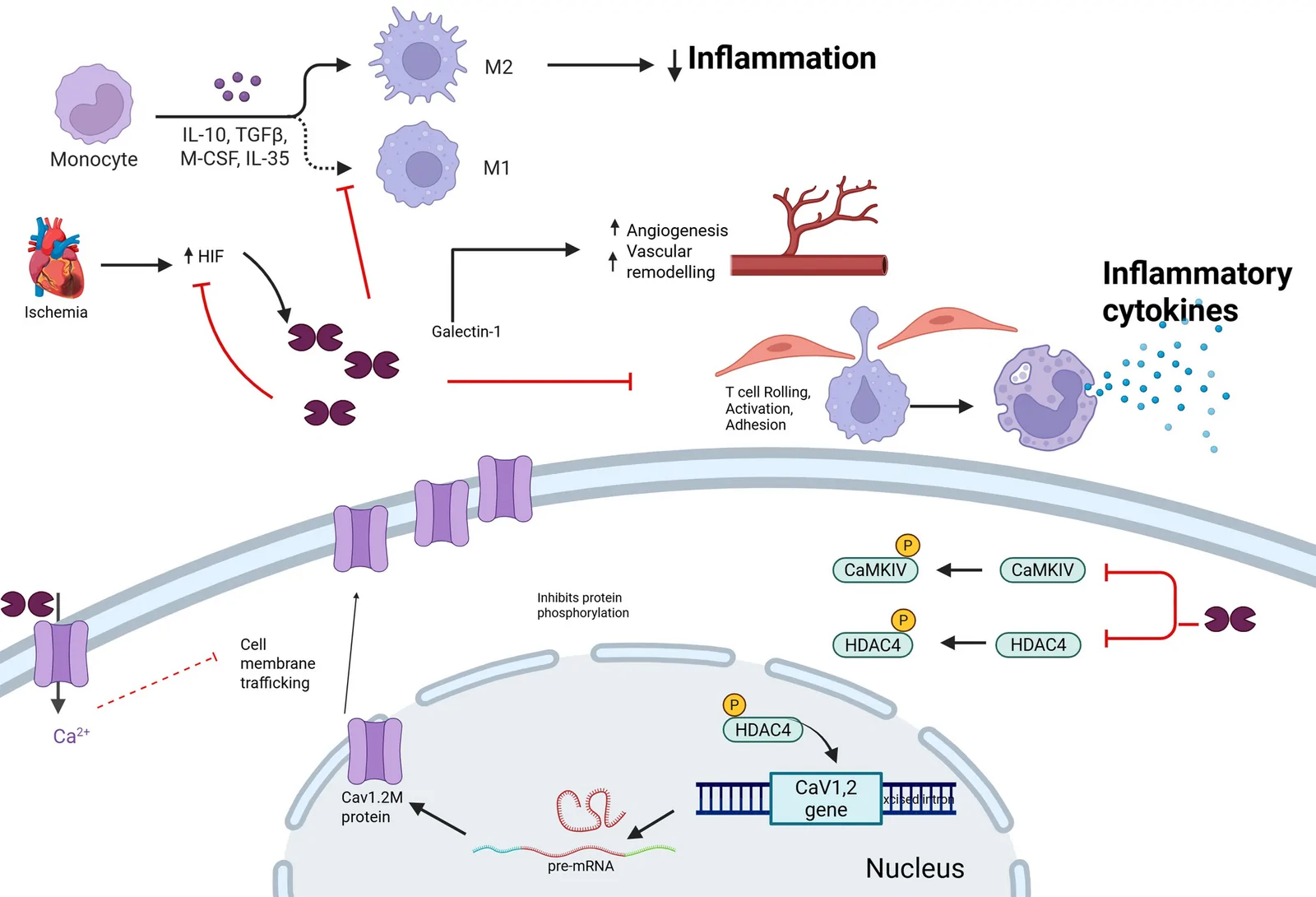
Cardioprotective roles of Galectin-1 in myocardial infarction: Schematic illustration of the multifaceted cardioprotective mechanisms of Galectin-1 in the ischemic heart. Following myocardial infarction (MI), Galectin-1 is upregulated as a result of hypoxia. Galectin-1 exerts immunomodulatory effects by promoting macrophage polarization toward the anti-inflammatory M2 phenotype while suppressing pro-inflammatory M1 activation. Concurrently, Galectin-1 inhibits T-cell rolling, adhesion, and activation, thereby reducing the release of pro-inflammatory cytokines and facilitating resolution of inflammation. In parallel, Galectin-1 promotes angiogenesis and vascular remodeling by enhancing endothelial cell proliferation, migration, and tube formation, contributing to improved perfusion and tissue repair. At the cellular level, Galectin-1 regulates L-type calcium channel (CaV1.2) activity in cardiomyocytes by modulating channel trafficking and expression through alternative splicing mechanisms. This results in reduced calcium influx and attenuation of calcium-dependent hypertrophic signaling pathways, including decreased phosphorylation of CaMKIV and HDAC4

During ischemia, HIF levels rise significantly, followed by a concomitant increase in both left ventricular and plasma Galectin-1 levels, suggesting its protective effects following cardiac events [27, 29, 79, 80]. In mouse models, Galectin-1 is upregulated in the heart seven days following acute MI, likely driven by proinflammatory cytokines. It is hypothesized that this upregulation plays a protective role by suppressing myocardial inflammation [42]. In rats, Galectin-1 has anti-apoptotic and anti-inflammatory effects in cardiomyocytes post-MI [81]. As early as 20 min post-MI, Galectin-1 level increases in the left ventricle, attributed to HIF-driven transcription. As ischemia progresses, dying cells in the infarct zone lose Galectin-1 expression, while surviving cells maintain or even increase Galectin-1 levels [80]. This early rise in Galectin-1 expression post-infarction is associated with low levels of apoptotic markers such as caspase-3, suggesting a cardioprotective role [80]. Notably, a subsequent clinical study found that aged patients with higher serum Galectin-1 levels exhibited a higher prevalence of hypertension and heart failure [82]. The possible mechanisms are depicted in Fig. 3.

## Resolution of inflammation

Galectin-1 is a prototypical β-galactoside-binding lectin widely recognized as a bona fide pro-resolving factor in the active resolution of inflammation [50, 83, 84]. Galectin-1 actively promotes the reprogramming of macrophages from pro-inflammatory to reparative, pro-resolving phenotypes, creating a microenvironment for resolving excess inflammation [83]. Consistent with these functions, Galectin-1 deficiency results in a failure to appropriately resolve inflammation [52, 85]. Rabinovich et al. demonstrated that local administration of Galectin-1 as an anti-inflammatory agent, effectively inhibited acute inflammation caused by bee venom phospholipase A2 in rat models. T lymphocyte capture, rolling and adhesion to target site endothelial cells is necessary for local inflammation, Galectin-1 modulates this process and causes T lymphocyte apoptosis [86]. In mice without Galectin-1, homing of T lymphocytes was significantly increased to lymph nodes and areas of inflammation [86].

Mice that were Galectin-1 deficient showed persistently elevated pro-inflammatory cytokine levels including IL-12 and diminished anti-inflammatory IL-10 [54]. By enforcing anti-inflammatory conditions, Galectin-1 helps to terminate the inflammatory response and restore tissue homeostasis. These immunomodulatory properties translate into pronounced cardioprotective effects in pathological conditions of the heart [83]. During cardiac infection such as Chagas disease caused by *Trypanosoma cruzi*, Galectin-1 protects the heart from pathogen-induced injury. In experimental Chagas disease, exposure of cardiac cells to Galectin-1 has significantly reduced parasite invasion and prevented infection-triggered apoptotic damage. Conversely, Galectin-1-deficient mice exhibit higher parasite burdens and increased mortality during *T. cruzi* infection [87, 88]. Similarly, in sterile myocardial injury such as MI, Galectin-1 is strongly induced in the infarcted heart to restrain post-infarction inflammation. Galectin-1-deficient mice subjected to MI develop exacerbated cardiac inflammation, with excessive macrophage and T-cell infiltration and a deficit of reparative regulatory T cells. Notably, therapeutic administration of recombinant Galectin-1 at the time of cardiac reperfusion significantly attenuated myocardial damage and improved post-MI ventricular remodeling [42]. Thus, by dampening deleterious inflammation sustaining immune responses, Galectin-1 potentially confers cardio protection following cardiac injury (Fig. 3).

### L-type calcium channel activity

L-type calcium channels are pivotal for calcium influx that drives excitation-contraction coupling in both vascular smooth muscle and CMs. Reduced calcium channel density has been linked to heart failure [89], and Galectin-1 has been shown to modulate calcium channels in CMs [90]. Intracellularly, Galectin-1 functions as a pre-mRNA splicing factor by participating in complexes that interact with other splicing components via weak protein-protein interactions [91, 92]. This role is particularly relevant in the regulation of CaV1.2 channels on cardiac cell membranes. Through a splice variant–specific mechanism, Galectin-1 reduces CaV1.2 channel density in CMs [93]. In resistance arteries and arterioles, CaV1.2-mediated calcium influx maintains myogenic tone whereas excessive L-type channel activity increases smooth muscle tone and raise blood pressure [94]. In the heart, CaV1.2 triggers cardiac contraction (via calcium-induced calcium release) and activates calcium-dependent transcriptional pathways. Galectin-1 plays a significant role in the modulation of these calcium channels as shown through Galectin-1 knockout mice that exhibited elevated arterial pressure (~ 20–30% increase in systolic/diastolic) with ~ 1.7-fold higher CaV1.2 levels in vascular muscle [95]. Notably, Galectin-1 binds directly to the calcium channel’s I–II loop; however, the strength of binding is weaker in alternatively spliced exon 9 variants [93, 95]. Galectin-1 binding reduces the functional surface expression of CaV1.2 and L-type calcium current density, thereby effectively mitigating calcium channel current. Additionally, Galectin-1 modulates alternative splicing of Cav1.2 M channels and inhibits key hypertrophic signaling pathways by decreasing the phosphorylation of proteins such as δCaMKII (Ca²⁺/calmodulin-dependent protein kinase II) and HDAC4 (histone deacetylase 4) [90]. It has been proposed that in acute MI, Galectin-1-mediated inhibition of calcium channels reduces calcium influx, thereby preventing excessive CM hypertrophy [90]. These mechanisms collectively contribute to the attenuation of myocardial hypertrophy, highlighting Galectin-1 as an emerging therapeutic target in cardiovascular diseases (Fig. 3).

## Angiogenesis

Freitag et al. demonstrated the pivotal role of Galectin-1 in fetal development through the promotion of vascular expansion [96]. In the adult heart cardiac homeostasis is governed by a multitude of pro-angiogenic processes, including inflammatory response, vascular regeneration, cardiac tissue remodeling, and exercise adaptation [70, 97]. By promotion of these pro angiogenic factors, Galectin-1 may facilitate repair and remodeling processes that benefit the heart in regulating angiogenesis makes it a potential key target in cardiovascular therapies [42]. Induced overexpression of Galectin-1 in spontaneously hypertensive rats significantly reduced their blood pressure [95]. While elevated Galectin-1 levels have been implicated in pathological conditions such as Abdominal Aortic Aneurysms, this may suggest that Galectin-1 role in angiogenesis maybe either maladaptive or protective given the tissue environment and context [98]. However, additional detailed research is warranted to decipher the molecular mechanisms underlying Galectin-1 driven angiogenesis in the heart tissue. The possible mechanisms are depicted in Fig. 3.

## Lipid profile

Furthermore, Galectin-1 was directly associated with lipoproteins such as LDL, VLDL, and cholesterol, while being indirectly correlated with HDL and HDL2 cholesterol levels [99]. Key inflammatory proteins, including interleukins and tumor necrosis factor (TNF), were positively associated with Galectin-1, reinforcing its role in metabolic inflammation and lipid metabolism [99]. Galectin-1 levels were increased in Diet Induced Obesity (DIO) mice at higher levels in subcutaneous fat as opposed to visceral fat. Leptin is a hormone secreted by adipose tissue and is involved in hunger suppression and lipolysis [100] where the Galectin-1 deficiency was associated with a loss of correlation between leptin and circulating fat mass [101]. Furthermore, Galectin-1 binds to lipoprotein A which is involved in artherogenesis. Lipoprotein A has several features, it closely resembles LDL in structure, binds Galectin-1 more strongly than LDL, and is expressed in the intima and media of atherosclerotic aortas [102]. These observations, demonstrate the role of Galectin-1 in the selective accumulation of lipoprotein A during artherogenesis [103]. This dysregulation could be a possible explanation for the increased Galectin-1 level in CAD/atherosclerosis patients. Logically, these observations lead to critical questions in Galectin-1 biology; (1) direct pro-atherogenic role, (2) insufficient level to protect from atherosclerosis, (3) tissue specific differential mechanisms, and (4) altered phenotype/subtype. These possibilities warrant further investigation.

### MI biomarker

Elevated circulating levels of Galectin-1 have been associated with increased severity of heart failure and left ventricular dysfunction, suggesting its potential as a biomarker for assessing post-MI complications and disease progression [89]. Heightened Galectin-1 concentrations in patients with eosinophilic fasciitis suggests a potential role in vascular impairment [104]. Galectin-1 is localized in the striated muscle of cardiac tissue, indicating its potential to be a local indicator of CM stress or injury [85]. In a study of patients with coronary artery disease awaiting a surgical procedure, higher pre-procedural Galectin-1 levels were associated with a worsened severity of coronary artery disease and had a higher chance of developing severe cardiac outcomes such as heart failure post-procedure [82]. Individuals with peripheral artery disease who have increased change of major adverse cardiovascular events, such as MI, were associated with higher levels of Galectin-1 [105]. These studies suggest that galectin-1expression is elevated during chronic inflammatory states, however the temporal relationship between Galectin-1 and these disease states is not fully elucidated. Galectin-1 elevation during chronic inflammation represents a cellular response in the pathogenesis of CAD. However, chronic Galectin-1 binding to atherogenic lipids worsens plaque formation in CAD patients [102, 103].

### Metabolic modulation

Galectin-1 plays a significant role in cardiac metabolism in physiological and stressful situations. In lipid metabolism, Galectin-1 enhances PPARγ expression and transcriptional activity, which induces lipid uptake and storage [32, 99]. Systemic increase in Galectin-1 promotes metabolic shift towards efficient lipid utilization that promotes heart health. Additionally, Galectin-1 influences glucose metabolism by facilitating beta-cell function and increasing glucose-stimulated insulin secretion [106]. In addition, Galectin-1 upregulates the expression of carbonic anhydrase IX and shift the Warburg effect to favor glycolysis [107]. This mechanism indicates that Galectin-1 combats acute MI where there is increased glucose levels and insulin resistance [108].

**Table 3:** Overview of Galectin-1-driven mechanisms for cardioprotection

Mechanism/Domain	Cardioprotective Effect	Details/Context	Reference
Anti-Inflammatory/Immune Regulation	Suppresses excessive inflammation and promotes resolution	Reprograms macrophages to anti-inflammatory (M2) phenotype and reduces IL-12, increases IL-10	(Dias-Baruffi et al. 2010) (Yaseen et al. 2020)
	Protects against inflammation induced heart damage	In Chagas disease: reduces parasite invasion and apoptosis and in MI: reduces T-cell/macrophage infiltration	(Benatar et al. 2015) (Seropian et al. 2013)
	Enhances regulatory T-cell response after MI	Galectin-1-deficient mice show impaired Treg recruitment and worsened inflammation post-MI	(Ou et al. 2022)
Post-MI Remodeling	Limits myocardial damage and improves ventricular remodeling	Recombinant Galectin-1 administration post-reperfusion reduces infarct size and preserves cardiac function	(Seropian et al. 2013)
L-Type Calcium Channel Modulation	Reduces calcium influx and cardiac hypertrophy	Binds splice variant of CaV1.2 channels, limits membrane trafficking, reduces calcium currents and HDAC4 activation	(Fan et al. 2019)
	Lowers arterial pressure in hypertensive models	Galectin-1-deficient mice showed increase BP and CaV1.2 expression and Galectin-1 therapy decreased BP in hypertensive mice	(Hu et al. 2018)
Anti-Hypertrophic Signaling	Inhibits hypertrophy-related pathways	Decreases phosphorylation of δCaMKII and HDAC4	(Fan et al. 2019)
Angiogenesis Modulation	Enhances endothelial proliferation, migration, and vessel formation, supporting vascular repair, remodeling, and blood pressure regulation	Promotes endothelial cell migration and proliferation during healing; also involved in fetal vascular development	(Tsai et al. 2018)
Biomarker Potential	Elevated Gal-1 levels reflect MI severity, heart failure, and vascular dysfunction	Associated with worse LV function, higher MACE risk, and vascular impairments	(Chou et al. 2020)
Metabolic Regulation	Supports metabolic adaptation by improving lipid use, enhancing insulin secretion, and promoting glycolysis during cardiac stress	Increases PPARγ levels, favors glycolysis, and enhances glucose-stimulated insulin secretion	(Baek et al. 2021) (Guda et al. 2022) (Sundblad et al. 2021)

### Translational significance and future directions

Galectin-1 has emerged as a promising cardioprotective protein due to its multifunctional roles in modulating inflammation, preserving vascular integrity, and regulating T-cell apoptosis. Despite its established relevance in various tissue healing contexts, the specific role of Galectin-1 in human cardiac pathophysiology, particularly under ischemic conditions remains insufficiently defined. Although Galectin-1 is known to influence key cardioprotective signaling cascades, such as VEGF-mediated angiogenesis and JAK/STAT pathway activation, most mechanistic insights are derived from studies in inflammatory or pulmonary models, underscoring the need for targeted cardiac investigations. Galectin-1 promotes angiogenesis and evades the immune system in cancer, and such knowledge could be extrapolated in cardiac cells following MI. Unfortunately, the studies that demonstrated Galectin-1-derived cardiac healing following MI and vascular healing are limited to small animal models [81, 109]. Also, the therapeutic safety and efficacy in human populations remain untested [43, 82]. Future directions should include investigating Galectin-1 as a circulating biomarker for assessing myocardial injury severity and predicting long-term outcomes following MI. Therapeutic strategies may involve antibody-based engineering or controlled delivery of exogenous Galectin-1 to infarcted tissue to promote localized angiogenesis, immune modulation, and myocardial regeneration. Furthermore, bioengineering approaches using induced pluripotent stem cells (iPSCs), mesenchymal stem cells (MSCs), or cardiac progenitor cells overexpressing Galectin-1 offer a compelling regenerative avenue. These engineered stem cells could potentiate cardiac repair, especially in heart failure models, though they warrant rigorous preclinical and translational evaluation. Lastly, Galectin-1 could be combined with other cardio-protective, anti-inflammatory therapies such as RNAse therapy which has shown promise in eradicating proinflammatory eRNA derived from ischemic cardiomyocytes [110]. Notably, no clinical trials to date have assessed Galectin-1-based therapies in cardiac patients. Individuals with inherently low endogenous Galectin-1 levels may be ideal candidates for such targeted interventions. Overall, Galectin-1 has significant roles in cardioprotective-relevant pathways and suggested safety in humans, therapeutic interventions using Galectin-1 remain promising.

## Conclusion

Galectin-1 is a strong cardioprotective protein that integrates inflammatory, metabolic, and angiogenic pathways that improve healing outcomes post-MI. Galectin-1 decreases inflammation by regulating immune cell phenotypes and cytokines. Metabolically, Galectin-1 enhances insulin secretion and supports energy-efficient adaptations under ischemic stress. In hypoxic environments, Galectin-1 promotes neovascularization and interact with endothelial cells for angiogenesis. High Galectin-1 levels are associated with impaired cardiovascular health post-MI, making Galectin-1 a potential biomarker. The characteristics of Galectin-1 highlight its therapeutic potential, and further research is needed to elucidate its mechanisms and use in cardiac therapy for humans.

## Data Availability

No datasets were generated or analysed during the current study.
